# Electrochemical Resistive DNA Biosensor for the Detection of HPV Type 16

**DOI:** 10.3390/molecules26113436

**Published:** 2021-06-05

**Authors:** José R. Espinosa, Marisol Galván, Arturo S. Quiñones, Jorge L. Ayala, Verónica Ávila, Sergio M. Durón

**Affiliations:** 1 Unidad Académica de Ingeniería Eléctrica, Universidad Autónoma de Zacatecas, Col. Centro, Av. Ramón López Velarde 801. Zacatecas, Zacatecas C.P. 98000, Mexico; 2 Unidad Académica de Ingeniería I, Ingeniería Mecánica, Universidad Autónoma de Zacatecas, Col. Centro, Av. Ramón López Velarde 801. Zacatecas, Zacatecas C.P. 98000, Mexico; 3 Unidad Académica de Ciencias Químicas, Universidad Autónoma de Zacatecas, Campus Siglo XXI, Edif. 6, Km 6 carr. Zacatecas-Guadalajara, Zacatecas C.P. 98160, Mexico; gavm001144@uaz.edu.mx (M.G.); arturochemistry.qfb@gmail.com (A.S.Q.); jayala69@uaz.edu.mx (J.L.A.); 4 Instituto Politécnico Nacional, Unidad Profesional Interdisciplinaria de Ingeniería Campus Zacatecas, Ingeniería Ambiental, Zacatecas C.P. 98160, Mexico; vavila@ipn.mx

**Keywords:** current relaxation, electrochemical HPV-16 DNA biosensor, potential relaxation, faradaic current

## Abstract

In this work, a low-cost and rapid electrochemical resistive DNA biosensor based on the current relaxation method is described. A DNA probe, complementary to the specific human papillomavirus type 16 (HPV-16) sequence, was immobilized onto a screen-printed gold electrode. DNA hybridization was detected by applying a potential step of 30 mV to the system, composed of an external capacitor and the modified electrode DNA/gold, for 750 µs and then relaxed back to the OCP, at which point the voltage and current discharging curves are registered for 25 ms. From the discharging curves, the potential and current relaxation were evaluated, and by using Ohm’s law, the charge transfer resistance through the DNA-modified electrode was calculated. The presence of a complementary sequence was detected by the change in resistance when the ssDNA is transformed in dsDNA due to the hybridization event. The target DNA concentration was detected in the range of 5 to 20 nM. The results showed a good fit to the regression equation $\Delta R_{total}\left(\Omega \right)=2.99\;\times \;\left[\mathrm{DNA}\right]+81.55$, and a detection limit of 2.39 nM was obtained. As the sensing approach uses a direct current, the electronic architecture of the biosensor is simple and allows for the separation of faradic and nonfaradaic contributions. The simple electrochemical resistive biosensor reported here is a good candidate for the point-of-care diagnosis of HPV at a low cost and in a short detection time.

## 1. Introduction

The International Agency for Research on Cancer (IARC) estimates that the number of cases of invasive cervical cancer in 2020 was 557,088 with more than 297,122 deaths caused by the disease. On incidence rates, cervical cancer was the third most common female cancer in the world in 2018 [1] and, after breast cancer and colorectal cancer, is the third leading cause of death in women worldwide [2]. HPV infection is a well-established cause of cervical cancer, and there is growing evidence that could be relevant in other anogenital cancers such as anal, vulval, vaginal, and penile, in addition to head and neck cancers [3]. Human papillomavirus infection is the most commonly transmitted disease in sexually active people around the world. The global prevalence of HPV infection is highest in women under 25 years of age, but in countries like Sub-Saharan Africa, Latin America, India, Mongolia, and China, where the incidence of cervical cancer is high, the prevalence of HPV increases [4,5,6,7,8]. HPV prevalence peaks in developed countries decrease in women after 35 years of age. However, in some Latin American countries, there is a second peak in prevalence in middle-aged women of 55 [4]. Prevalence is also related to the grade lesions; thus, the prevalence of HPV 16/18 in women with normal cervical cytology is 3.9% and increases to 25.8%, 51.9%, and 69.4% in women with low-grade lesions, high grade lesions, and cervical cancer, respectively [9].

Worldwide, the most common types of HPV in cervical-uterine cancer are: 16 (57%), 18 (16%), 58 (5%), 33 (5%), 45 (5%), 31 (4%), 52 (3%), and 35 (2%) [10]. Types 16, 18, and 45 represent a greater or equal proportion of cervical cancer infections. Highly sensitive, accurate, and specific methods are crucial for clinical diagnosis and prognosis. Current methods based on DNA hybridization meet the above requirements; however, it should be imperative that the hybridization process be evaluated by means of an adequate compact device, such an electrochemical biosensor.

DNA detecting sensors have gained importance in recent decades as diagnostic tests for genomics and as early detection tools for cancer or for other diseases [11]. Many DNA biosensors have been described in the literature based on diverse principles, such as electrochemistry [12,13,14] and optics [15,16,17,18]. Optical detection is based on fluorescence spectroscopy, which uses a laser beam as a source for fluorophore-tagged DNA excitation, and photomultipliers or charge-coupled devices for the detection of emitted light. However, optical systems require labelling with fluorescent molecules and relative expensive instrumentation, limiting their portability for point-of-care applications [19].

In contrast, biosensor systems based on electrochemical techniques, such as chronoamperometry [20], capacitance [21,22], and electrochemical impedance spectroscopy (EIS) [20], have been shown to be of great utility due to their sensibility and low cost [23,24]. By detecting changes that occur during hybridization at the interface between a DNA functionalized electrode and a conductive target analyte solution, electrochemical techniques have the potential to provide real-time measurement, label-free sensing, and more portable detection platforms [25].

Many EIS biosensors have been based on a DNA hybridization event that results in changes in the impedance or surface charge of a DNA-modified working electrode. An impedance spectrum analyzer extracts the real and imaginary components of the biosensor impedance, and the impedance variation can be correlated with immobilized nucleic acid properties and with the concentration of target in a sample [26,27]. A major advantage of EIS is that detection can be performed label free, i.e., the changes in the electrical properties of the electrode surface arise from the interaction with the target molecule alone [28]. Even though this technique is highly sensitive, this advantage sometimes limits its application as a result of being liable to respond to interferences, too. Furthermore, genosensors that rely on electrochemical impedance spectroscopy require a long data-acquisition time [29]. EIS has been successfully replaced by other less sophisticated electrochemical techniques [30], such as differential pulse voltammetry [31], chronoamperometry [31,32,33], chronopotentiometry [34], or switching DNA [35]. A structure-switching probe operates via the alteration of distance of the redox labels from the electrode caused by target-induced structure switching, representing a significant advance of using minimal reagents with working steps, a simplified setup, cost efficiency, high sensitivity, and excellent compatibility with miniaturization potential [35].

After the discovery of the electrically induced conformation switching of DNA oligonucleotides on metal surfaces, switchable biosurfaces have been used successfully for the detection of DNA and protein targets with high sensitivity [36,37]. Typically, this methodology has been used in optical sensors; however, very little literature has been found on switchable biosurfaces applied to electrochemical sensors.

Previously, we reported an electrochemical method for HPV-16 sensing based on potential relaxation [38], where the results showed the relevance on sensing measurements of the double layer discharge of DNA-modified gold electrodes. As a continuation in this area of research and with the aim of improving the electronics and time of detection, this work describes a system for sensing DNA related to human papillomavirus (HPV), based on the “current relaxation” (CR) method. This method is related to the conformational properties of DNA bioelectrodes to obtain the change of the charge transfer resistance of a redox indicator couple due to the hybridization of ssDNA immobilized on screen-printed electrodes (SPE) in a short time (750 µs) and by using only one potential step. In the CR method described here, the relaxation current and potential were simultaneously measured by employing an external electrical capacitor. This single potential and current relaxation measurement allows for computing from the Ohm’s law the charge transfer resistance of the DNA-modified electrode. The advantage of the CR method with respect to traditional impedimetric methods used in DNA biosensors is that by using a very simple electrical architecture, the charge transfer resistance on the DNA-modified electrode can be directly evaluated, thus avoiding the use of computational algorithms for fitting an equivalent circuit to separate the current transfer components (faradaic and nonfaradaic).

## 2. Materials and Methods

### 2.1. Chemicals and Reagents

**Figure 1:**
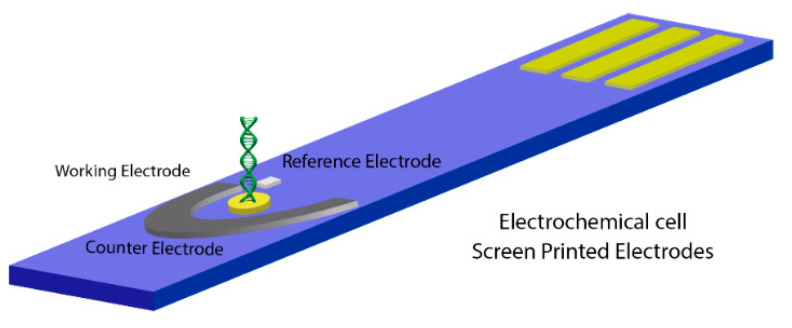
Schematic drawing of the electrochemical cell.

Electrochemical measurements were performed by using an SPE of the dimensions 3.4 × 1.0 × 0.05 cm (Figure 1), composed of an Au disk with an immobilized layer of DNA probe as the working electrode (4 mm diameter), a Ag/AgCl/NaCl reference electrode (E = 242 mV NHE), and a carbon counter electrode. In this work, all the reported potential values are referred to the Ag/AgCl reference electrode, except when indicated. The hybridization studies of the bioelectrode were carried out by using a potential step chronoamperometry, and in order of comparison, complete electrochemical impedance measurements of the bioelectrode were made to characterize the hybridization process. All electrochemical measurements were made in a (2 mM K_4_(Fe(CN)_6_) + 2 mM K_3_(Fe(CN)_6_) in (50 mM PBS + 100 mM K_2_SO_4_) solution (pH 7.4), with an ionic strength of 447 mM.

The oligonucleotide stock solutions were prepared with 20 mmol L^−1^ Tris-HCl buffer, pH 7.4 solution (Tris) and kept frozen. The 30-base oligonucleotide sequences HPV-related used in the present study were:

DNA probe = (5′-HS(CH2)_6_GTCATTATGTGCTGCCATATCTACTT-CAGA-3′);
DNA complementary target = (5′-TCTGAAGTAGATATGGCAGCACATAATGAC-3′);
DNA single-based mismatch target = (5′-TCTGAAATAGATATGGCAGCACATAATGAC-3′).

The type-specific HPV oligo probe corresponds to a region of heterogeneity within the HPV L1 that is flanked by the GP5+/GP6+ primer pair, and it was designed by Jacobs et al., who determined the specificity of this oligonucleotide sequence [39]. The thiol ending of the DNA probe forms chemical bonds with the gold surface atoms; as a consequence, any leaching of DNA molecules was observed during experiments.

### 2.2. DNA Probe Immobilization and Hybridization with DNA Target

The SPE were washed with water and subsequently electrochemically cleaned within 0.5 M H_2_SO_4_ by using cyclic voltammetry, scanning the potential between −0.05 V and +1.1 V for approximately 60 cycles until no further change in the voltammogram was obtained.

The immobilization of probe DNA was obtained by depositing on the gold electrode a drop of 10 µL of a 1 µM ssDNA solution in immobilization buffer for 1 h at room temperature. The DNA immobilization buffer consisted of 0.8 M phosphate buffer (PB) + 1.0 M NaCl + 5 mM MgCl_2_ + 1 mM EDTA, pH 7.0. After immobilization, the electrode was sequentially washed in the following solutions to eliminate the redundant probes at: immobilization buffer, 200 mM PB, 10 mM PB, and finally, 10 mM PB + 10 mM EDTA to remove any remaining magnesium ion. To ensure a uniform layer on the gold surface and to avoid nonspecific interactions of oligonucleotides, the electrodes were subsequently electrochemically cleaned by scanning the potential between −0.4 V and +0.4 V for approximately 50 cycles until no further change was observed.

For hybridization experiments, 10 μL of a 20 nM solution of complementary target DNA in PBS, pH = 7.4, were drop coating deposited on the Au/ssDNA probe electrode and incubated at 37 °C for 1 h. The optimization results indicate that no additional time is required to obtain a maximum in the hybridization event, since no further changes were observed in EIS spectra for times greater than 1 h.

The DNA immobilization and hybridization were analyzed using an Fe(CN)_6_^4−^/Fe(CN)_6_^3−^ redox couple. The biosensing analytical performance of the DNA bioelectrode was studied with complementary target concentrations of 20, 15, 10, and 5 nM. The detection limit was calculated as three times the standard deviation of the blank sample measurement. For specificity tests, solutions of complementary and single-base mismatch sequences were used; the hybridization response of each was compared by using a Student’s *t*-test.

### 2.3. Electrochemical Measurements

Electrochemical detection was performed by applying a potential step from the open circuit potential (OCP) to a potential of 30 mV with respect to the OCP value during 750 µs. Then, the system was relaxed back to the OCP, and the voltage and current discharging curves were registered for 25 ms. From these discharging curves, the potential relaxation and current relaxation were measured, and the charge transfer resistance ($R_{total}$) through the DNA-modified electrode was calculated. The presence of a complementary sequence is detected by the change in resistance when the ssDNA is transformed in dsDNA due to the hybridization event.

In order to compare the $R_{total}$ values obtained from the relaxation curves, electrochemical measurements of the modified electrodes were performed in PBS solution (pH 7.0) by using the EIS technique and obtaining the resistance values by a nonlinear least squares fitting (CNLS) of the experimental impedance data. The impedance was measured over the frequency range from 100 kHz to 100 mHz, with a 10 mV a.c. amplitude voltage superimposed on a d.c. bias of 30 mV with respect to the open circuit potential, which corresponds to the formal potential of the Fe(CN)_6_^4−^/Fe(CN)_6_^3−^ redox couple. $R_{total}$ was measured before then after DNA hybridization. The solution resistance ($R_{s}$) was measured using the same technique with an excitation frequency of 100 kHz. The potential and current relaxation experiments and EIS were carried out by using a Reference 600 Gamry potentiostat/galvanostat.

### 2.4. The Current Relaxation Method

**Figure 2:**
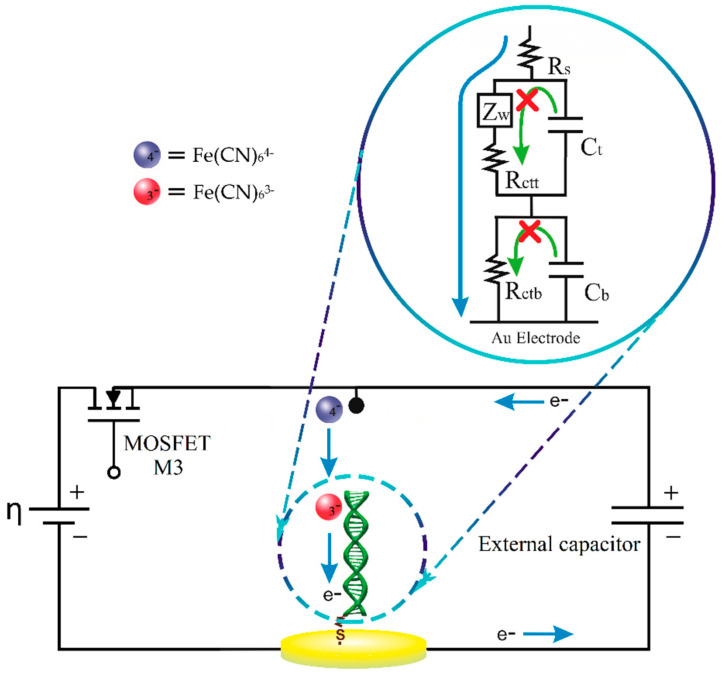
The electric current relaxation method.

In the current relaxation methodology, the contribution of double layer discharging currents and the time constants related to capacitive and resistive elements present on the surface of the electrode Au/DNA [38] are minimized due to the operation of the electric circuit used in the measurements. The use of the external electric capacitor as shown in the scheme of Figure 2, allows us to measure the voltage and the electric current in instants of time where the double layer relaxation currents are not present on the surface of the electrode.

**Figure 3:**
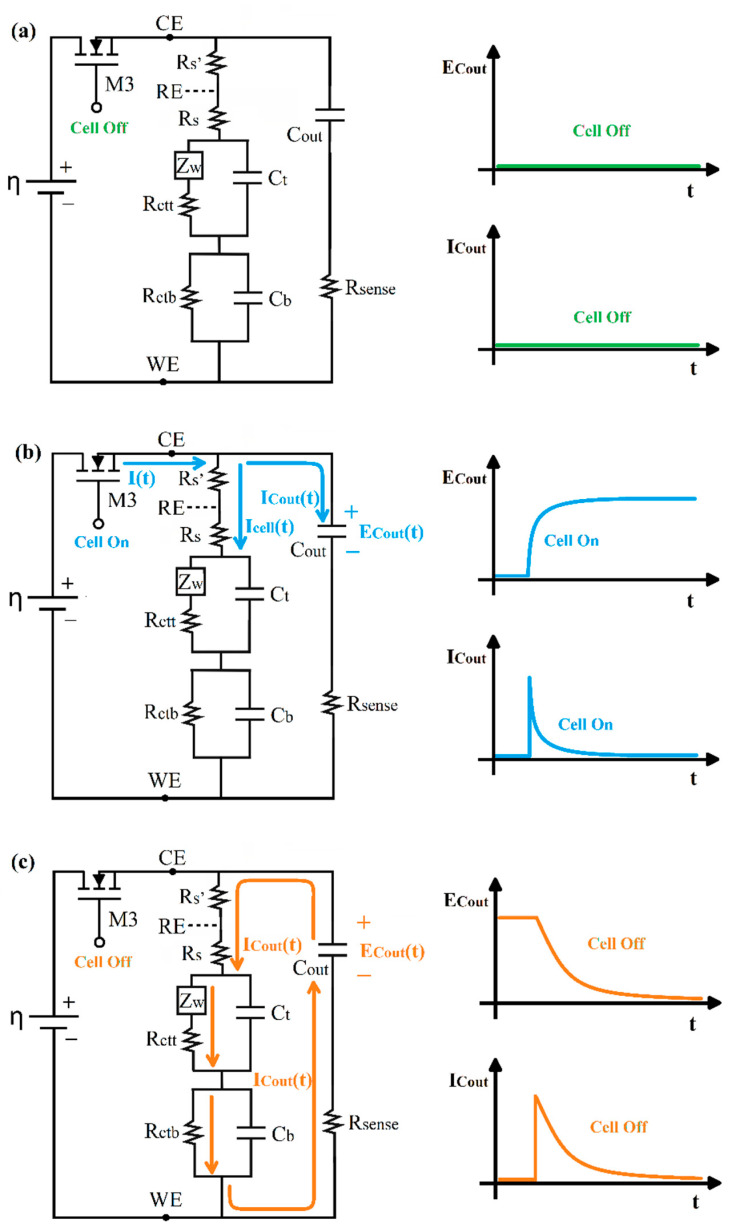
Proposed electrical diagram to determine the current relaxation: (**a**) external capacitor discharged and double layer in equilibrium; (**b**) external capacitor and double layer charging stage; (**c**) external capacitor and double layer discharging stage.

In Figure 3, the electrical diagram of the CR method is further described, and the working electrode (WE) corresponding to the DNA/Au system is represented by an equivalent circuit (EC), previously published, comprised of two closed loops related to two relaxation processes [38]. The top loop is associated with the DNA region far from the metallic electrode and includes a Warburg element ($Z_{W}$) that describes the diffusion impedance of the redox indicator anions between the solution and the DNA electrode and a charge transfer resistance $R_{ctt}$ in parallel with the capacitor $C_{t}$. The bottom loop includes only the resistance $R_{ctb}$ and capacitor $C_{b}$ related to the electrochemical processes occurring in the DNA region next to the Au electrode. $R_{s}$ corresponds to the solution resistance, and $C_{out}$ represents the external electrical capacitor.

For the minimizing of the double layer discharging current, the electrical external capacitor ($C_{out}$) is incorporated in parallel with the electrochemical cell. At a time equal to zero, the cell is off because MOSFET transistor (M3) is in the “cut off” region operation. The electrode Au/DNA is in a state of electrochemical equilibrium and no potential is applied on (Figure 3a); at this condition the electrical potentials in the circuit can be expressed by

(1) $$\;E_{R_{s}}=E_{C_{dl}}=E_{R_{ct}}=0\;V$$

where $E_{R_{s}}$, $E_{C_{dl}}$, and $E_{R_{ct}}$ correspond to the rest potentials of the WE equivalent circuit elements.

At a time greater than zero, the cell turns on, the M3 transistor turns on, it enters in its saturation region, and a direct current electrical potential ƞ is applied (Figure 3b). At this stage, two major electric currents are generating. The first is circulating in the electrochemical cell, which corresponds only to the faradaic process. The second electrical current is the charging current of the external capacitor, which by nature decreases exponentially with respect to the time $I_{Cout}\left(t\right)$. The sum of the two electric currents corresponds to the total current demand by the detection system, as described by the equation

(2) $$I\left(t\right)=I_{cell}\left(t\right)+I_{Cout}\left(t\right)$$

The external capacitor charging current over time is governed by the expression

(3) $$I_{Cout}\left(t\right)=\frac{\eta }{R_{sense}}e^{-\frac{t}{\tau_{out}}}$$

where $\tau_{out}$ is the time constant of the external capacitor $C_{out}$ in the loading step, which may be expressed as

(4) $$\tau_{out}=R_{sense}C_{out}$$

At a time greater than five time constants $\left(t>5\;\tau_{out}\right)$, the charge in the external capacitor is practically complete; then, the cell is turned off with the M3 transistor (Figure 3c), and the applied potential is cut off to the electrochemical cell and the $C_{out}$. At this time, the external capacitor starts discharging through a global resistance $R_{total}$ represented by the equation

(5) $$R_{total}=R_{sense}+R_{s\prime }+R_{s}+R_{ctt}+R_{ctb}$$

This equation indicates that $R_{total}$ corresponds to the series sum of all resistances of the cell and the external electrical circuit.

At this step, the voltage and current in the external capacitor will decrease with respect to time according to the following equations, respectively:

(6) $$E_{Cout}\left(t\right)=\eta e^{-\frac{t}{\tau_{discharge}}}$$

(7) $$I_{Cout}\left(t\right)=\frac{\eta }{R_{total}}e^{-\frac{t}{\tau_{discharge}}}$$

where $\tau_{discharge}$ is the discharge time constant of the external capacitor, which can be calculated from the equation

(8) $$\tau_{discharge}=R_{total}C_{out}$$

Solving for η from Equation (6) and substituting it into Equation (7), we obtain the following expression:

(9) $$I_{Cout}\left(t\right)=\frac{E_{Cout}\left(t\right)}{R_{total}}$$

or

(10) $$R_{total}=\frac{E_{Cout}\left(t\right)}{I_{Cout}\left(t\right)}$$

According to Equation (5), a change in the total resistance (Δ$R_{total}$) is attributed only to the change in the charge transfer resistance ($R_{ct}$), because the experimental design implies that the solution resistances (*Rs′*, *Rs*) and sense resistance ($R_{sense}$) are constants in the biosensor. Therefore, a change in charge transfer resistance (Δ$R_{ct}$) is equivalent to the change Δ$R_{total}$; this is the detection parameter of the resistive biosensor that indicates the DNA hybridization in the electrode surface. With the use of Equation (10), it is thus possible to measure the charge transfer resistance at a given instant of time without the need to use more sophisticated techniques, such as electrochemical impedance spectroscopy.

The potential used in the first part of the experimental sequence was selected such that the external capacitor could be charged while it provides enough electrical current to the HPV DNA/Au electrode, maintaining the system kinetically limited. As a consequence, the potential and current discharge provide exclusive information concerning the HPV DNA sequence attached to the Au surface and their electrochemical changes, which are related to the hybridization process and associated redox reactions.

### 2.5. Sensing Circuit

**Figure 4:**
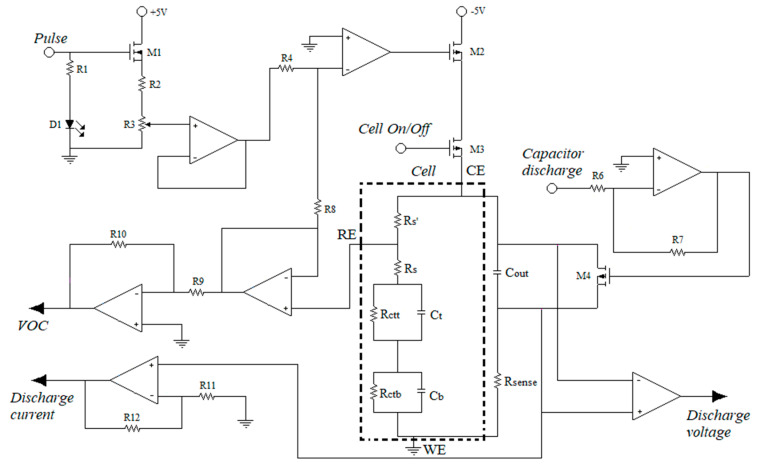
Schematic block electronic architecture of the DNA biosensor.

The electronic architecture of the HPV biosensor is based on a three-electrode system and other elements that allow the proper performance of the relaxation current methodology described in the previous section, as well as the functions of applying the voltage step to the cell, reading the redox electric current, and providing the analog signals for later digitization. This architecture is constituted of six main parts and is shown in Figure 4.

The general topology of the biosensor to evaluate the resistance change depends on three readout interfaces (*discharge current, VOC, and discharge voltage*) and three control interfaces (*Pulse, Capacitor discharge, and Cell On/Off*). The *discharge current* stage is responsible for converting faradaic electrical current to voltage through the $R_{sense}$ resistor, amplifying and transmitting the signal through an output channel for further processing. *Discharge Voltage* measures the voltage difference in the external capacitor. *VOC* measures the open circuit voltage of the electrochemical cell. *Cell On/off* is a digital input that activates the MOSFET (M3) for interconnection between the cell and the biosensor. Through the M3, the voltage and current are applied to the electrochemical cell. The *Pulse* input is responsible for activating and applying the direct current potential to the working electrode. The pulse amplitude is adjusted with the resistor R3. *Capacitor discharge* has the function of short circuiting the external capacitor to perform a new measurement. All these input and output functions are controlled by an external microcomputer (Raspberry Pi 4).

## 3. Results and Discussion

### 3.1. Step Potential

**Figure 5:**
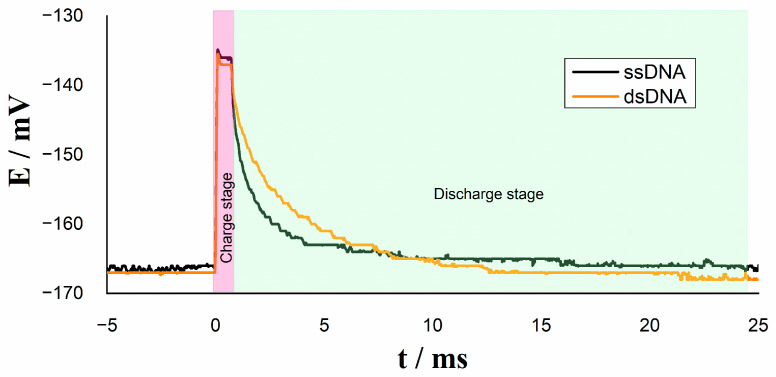
Relaxation voltage without external capacitor $C_{out}$ on Au/ssDNA and Au/dsDNA electrodes.

To evaluate the performance of the resistive sensor, the relaxation voltage signals of ssDNA and dsDNA in a step potential were registered. Analyzing the decaying voltage in each pulse, as previously reported, a closer inspection of double-layer responses revealed that the potential curves were in fact composed of two processes related to two different relaxation times in ssDNA and dsDNA in the absence of any external capacitor [38] (Figure 5).

**Figure 6:**
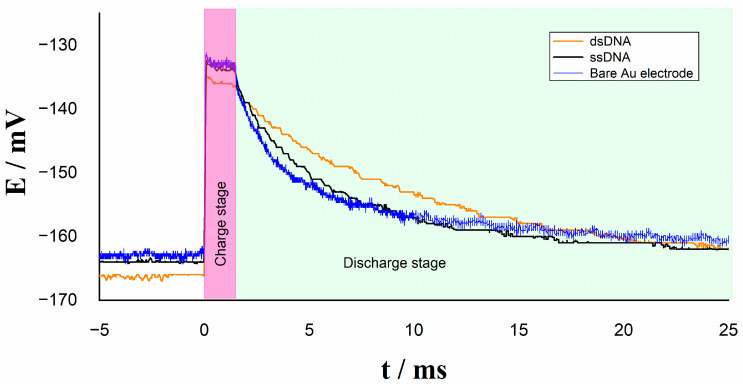
Relaxation voltage with external capacitor $C_{out}$ on different electrodes.

Under the same experimental conditions, a new experiment was performed by using an external capacitor in the measurement system (Figure 6). In this case, we can observe the presence of only one relaxation time on bare Au, ssDNA, and dsDNA electrodes; this result is the effect of the external capacitor to minimize the charge capacitors’ effect corresponding to the DNA/Au electrode surface. The relaxation time is now directly attributed to the product of the resistance $R_{total}$ with the external capacitor $C_{out}$ as expressed in Equation (8).

**Figure 7:**
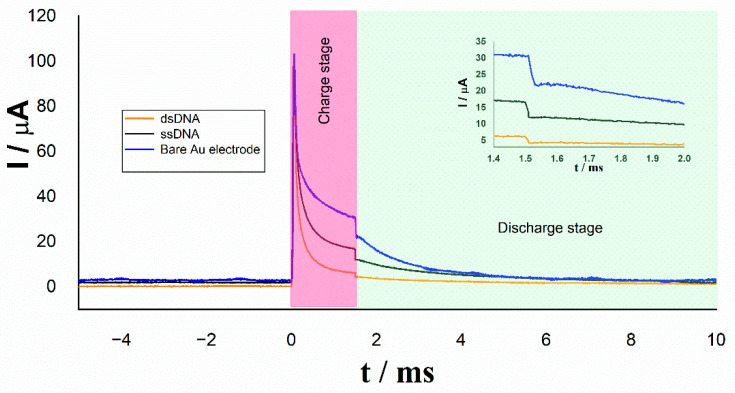
Relaxation current with external capacitor $C_{out}$ on different electrodes.

Analyzing the faradic and nonfaradaic current in each pulse by using the external capacitor as shown in Figure 7, at the first stage where the potential is being applied, we can appreciate the electric current demanded by resistor $R_{total}$ (stationary state) and capacitor $C_{out}$ (transient state). When the potential step is canceled, we can see an abrupt change in current curve due to the switching of discharge current created by the M3 transistor in its cut-off operating region. At this time, the decaying electric current is related to the resistance $R_{total}$, and the potential difference present on the capacitor $C_{out}$ is expressed in accordance with Equation (9).

The inset in Figure 7 highlights the decrease in electric current due to the immobilization of the single strand DNA deposited on it (black line) with respect to the bare gold electrode (blue line). A greater decrease in current can be appreciated in the electrode after the hybridization of the single strand DNA with the target complementary (orange line).

The current decrease is interpreted as an increase in resistance $R_{total}$ on the electrode surface due to the electrostatic repulsion between negative charges of DNA backbone and the ions of redox couple Fe(CN)_6_^4−^/Fe(CN)_6_^3−^, which results in a barrier for the interfacial electron transfer.

According to the experimental behavior, the $R_{total}$ evaluation from the voltage and current measurements, can be done with a sufficient accuracy under four times the relaxation constant period. The electronic design of the CR biosensor allows for fulfilling this condition by measuring such parameters at the very beginning of the discharge stage. As a result, the measure of the voltage on the external capacitor and the electric current flowing in the electrochemical detection system is carried out at a time near 1.5 ms, and the value of $R_{total}$ is instantly obtained by application of the Ohm’s law.

In Table 1, the values of the relaxation current and resistance obtained from the curves of Figure 7 are compared for the different electrodes. The significative changes in the measured values of *I_relax_* and *R_total_* when the working electrode is modified from bare Au to ssDNA/Au and to hybridized dsDNA/Au can be appreciated.

The difference between the $R_{total}$ value of at the ssDNA/Au and the dsDNA/Au surfaces were used as the measurement signal to determine the DNA specific sequence hybridization related to HPV type 16, as expressed in Equation (11):

(11) $$\Delta R_{total}=R_{total\;dsDNA}-R_{total\;ssDNA}$$

Finally, in order to minimize the signal variation ratio (SVR) upon hybridization among different experiments, the percentage change in $R_{total}$ was determined according to the following equation:

(12) $$\Delta R_{total\%}=\frac{R_{total\;dsDNA}-R_{total\;ssDNA}}{R_{total\;ssDNA}}\times 100\%$$

One of the main characteristics of the biosensor proposed in this work with respect to other similar detection systems lies in the ease with which the CR resistive biosensor suppresses the interference effect of the nonfaradaic currents of the electrochemical system by using an external capacitor for background subtraction. To date, few reports have applied hardware subtraction of background signals in chronoamperometric techniques to measure the faradaic current in DNA sensors. Mark D. Holtan et al. [40] reported a new electrochemistry hardware that considerably suppresses nonfaradaic currents through real-time analog subtraction during current-to-voltage conversion in the potentiostat. According to these authors, an electronic structure called differential potentiostat (DiffStat) removes capacitance currents in chronoamperometry applied to a DNA monolayer. On the other hand, a recent report has demonstrate the potential advantages of using chronoamperometry to measure the change in electrokinetics by employing a direct fitting of chronoamperometric data to remove the background digitally [41]. The main difference between such methodologies and the CR, relies in the measurement times. In the proposed sensor only one point of chronoamperometry curve is required to evaluate the charge transfer resistance, requiring measuring times less than 1 ms. In constrast, in the two aforementioned techniques, the complete current curve in both the transient and stationary regions must be analyzed; as a consequence, large global performing measurement times from 10 s to 100 s are required for a complete measurement [42].

### 3.2. Analytical Performance

**Figure 8:**
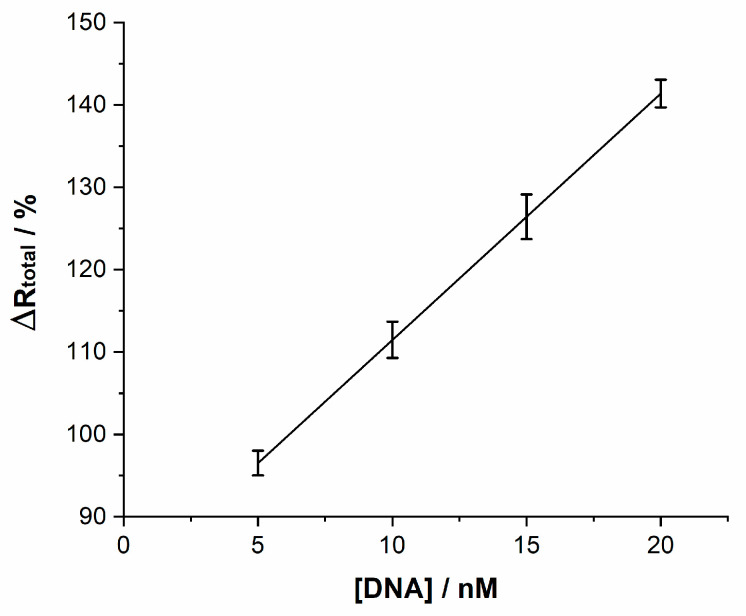
Sensitivity of the DNA biosensor at different concentrations of DNA.

The analytical performance of the DNA biosensor was studied by using different concentrations of the complementary sequence. Figure 8 depicts the linear relationship between the percentage in $\Delta R_{total}$ change at different concentrations of target HPV-16 DNA. As can be seen, the percentage change increases with the target HPV-16 DNA concentration. A dynamic linear range from 5 nM to 20 nM for the target DNA was achieved. The results fit to the regression equation $\Delta R_{total}\left(\Omega \right)=2.99\times \left[\mathrm{DNA}\right]+81.55$, the square of the linear correlation coefficient was 0.955, and a detection limit was estimated to be 2.39 nM at 3σ (σ = standard deviation of blank signal). These resistive results confirm an effective detection of the hybridization of the complementary target HPV-16 DNA on the sensor at the studied concentration range. The error bars shown in the plot correspond to the dispersion of resistance values of at least three measurements.

**Figure 9:**
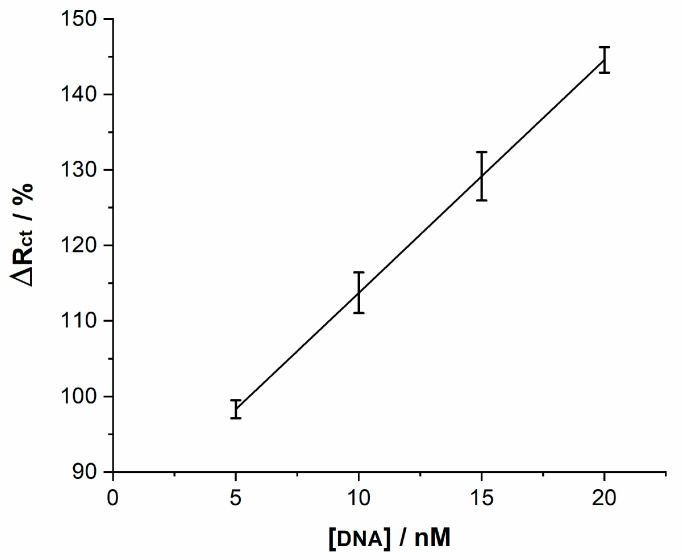
Sensitivity of the DNA biosensor with EIS electrochemical technique at different DNA concentrations.

The analytical performance of the biosensor was compared with electrochemical impedance measurements of hybridization on the probe DNA electrodes at the same concentrations of target oligonucleotides (see Figure 9). Similarly, a linear increase of $\Delta R_{ct}$ with the concentration of complementary HPV-16 DNA that fits to the equation $\Delta R_{ct}\left(\Omega \right)=3.08\times \left[\mathrm{DNA}\right]+82.88$, $R^{2}=0.933$ and a detection limit of 2.41 nM was also observed. In order to obtain the change in $R_{ct}$ which relates to hybridization, the experimental impedance spectra were fitted to an equivalent circuit previously reported [38].

From the slopes of the two fitting linear equations of the calibration curves, it can be appreciated that the sensitivity to complementary HPV DNA are similar between relaxation current sensor measurements and electrochemical impedance spectroscopy results. However, the relaxation current technique has the advantages of a shorter measurement period and a simpler electronic architecture than those of EIS.

A true comparison between the different types of electrochemical DNA biosensors for the diagnostic of HPV is very difficult due to the great diversity of techniques and methodologies reported for the detection of HPV. However, Table 2 shows a comparison between various types of biosensors applied to the diagnosis of HPV-16, 45, and 18. The advantage shown by the proposed biosensor with respect to other published methods lies in the optimization of the detection system which does not require an exhaustive modification of the electrode (use of nanosheets, magnetic beads, carbon nanotubes, etc.) to obtain a limit of detection similar to other electrochemical techniques. Furthermore, the electronic architecture of the biosensor is very simple, and the detection time for a measurement is shorter than those observed in other reported HPV biosensors.

### 3.3. Specificity of the DNA Biosensor

The hybridization specificity of the biosensor was evaluated by using two different target DNA: complementary DNA sequence (C) and single-base mismatch (SBM).

**Figure 10:**
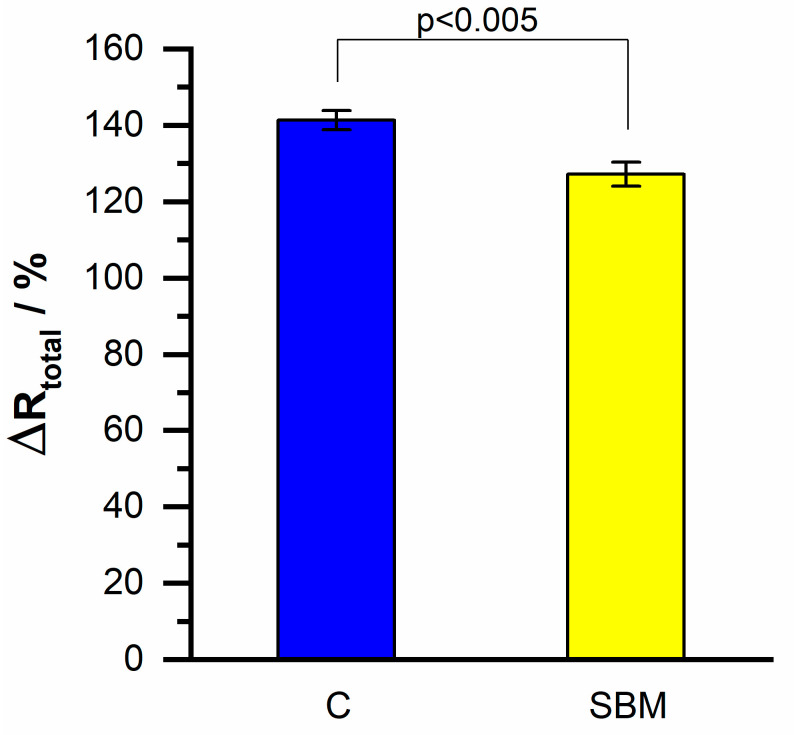
Charge transfer resistance values of hybridization with complementary (C) and single-base mismatch target (SBM) in resistive DNA biosensor.

Figure 10 shows the comparison of the change in charge transfer resistance on the resistive sensor for the detection of 20 nM of complementary HPV-16 DNA against the measurement of hybridization with a single-base mismatch target of the same concentration. A difference near 14 percentage units in the measured $\Delta R_{total}$ can be observed, which, examined with the Student’s *t*-test, indicates a significant difference between the two obtained values. This can be interpreted as an evidence of the high specificity of the resistive DNA biosensor regarding the detection of HPV-16 DNA.

## 4. Conclusions

In this work, a sensor prototype was reported that showed an acceptable performance towards the evaluation of the change of charge transfer resistance associated with the hybridization of ssDNA with its complementary target HPV-16 DNA.

A very important advantage that the “current relaxation” technique shows compared to the EIS technique is the detection time. Typically, in conventional EIS experiments employing a 100 kHz–10 mHz range of frequency, the complete spectrum is captured in an average length of time of 35 min. In contrast, the detection timeout in the resistive DNA biosensor was 750 μs, which makes it a new fast technique compared to the traditional EIS applied to DNA biosensors.

As it is known, the “only one frequency” EIS is a common time-saving procedure to obtain the charge transfer resistance in electrochemical sensors. However, in this approach, while it is only necessary to apply a unique AC frequency to obtain the values of the electrochemical parameters, the reliability depends on the equivalent circuit used to simulate the system by using the nonlinear square fitting procedure. In the case of complex electrodes, such as the HPV-16/DNA/Au ensemble used in this work, the electrochemical behavior requires an improved equivalent circuit that includes two parallel RC arrangements, different from a single Randles circuit [38]. As a consequence, the “only one frequency” approach is not completely adequate to evaluate the fitted parameters of these kind of sensing electrodes due to the presence of the two characteristic time constants of the system.

The results obtained with the sensor reported here for HPV-16 DNA detection mean that this type of biosensor can be applied in bioelectrodes modified with multilayers of another type of recognition biomolecules to identify different analytes or DNA sequences related with other diseases or biological systems, where the classical electrochemical techniques, such as EIS, are difficult to implement due to their costs or technical issues.

In relation to DNA biosensors based on chronoamperometry, the resistive biosensor imposes certain advantages due to the ease of separation of faradic from nonfaradaic currents, avoiding the use of complex algorithms to separate the current contributions.

Furthermore, the electronic architecture proposed here for the sensor that operates only with direct current is simpler than that of the EIS methodology, despite the simplifications implicit in the use of one frequency impedance measurement. As a consequence, the detection timeout required to complete an electrochemical measurement in the resistive sensor, less than 1 millisecond, makes the current relaxation technique competitive with other methodologies, such as the EIS one frequency mode, that require several milliseconds to be carried out.

Due to the sensing procedure based in direct current, the architecture of this genosensor is a simple one since it does not need electronic noise generation nor long computer memory facilities to store the data generated by experiments. As future work, in an advanced stage in the construction of the biosensor, a low-cost microcontroller must be integrated to cover the specific needs for the digital processing of the acquired signals. This complete embedded system will allow the registration of medical test results and could share them instantly on a Wi-Fi network and enable screen display facilities. Additionally, to improve the sensitivity of the sensor, it is necessary to modify the surface structure of the substrate electrode by employing nanoparticles of different materials or charge transfer mediators in order to achieve a detection limit near 10^−12^ molar, which is of clinical value.

The displayed results suggest that the electrochemical resistive DNA sensor reported can be regarded as a good candidate for HPV-16 point-of-care medical diagnosis.

## Figures and Tables

**Table 1:** Comparison of relaxation currents and total electrical resistance for different electrodes at t ≅ 1.5 ms.

	*I_relax_/*µA	*R_total_*/kΩ
**Bare Au electrode**	22.2	1.3
**ssDNA**	11.9	2.4
**dsDNA**	4.3	6.6

**Table 2:** Comparison of electrochemical DNA biosensors for HPV.

HPV Type	Technique	Sensor Platform	Detection Limit	Response Time	Ref.
HPV-16	DPV	PGE	1.49 nM	40 s	[43]
HPV-16	SWV	Carbon surface/chitosan	4 nM	10 s	[44]
HPV-16	EIS, SWV	Paper base/G-PANI	2.3 nM	17 min, 15 s	[45]
HPV-16	CV	GCE/CNO	0.50 nM	7 min	[46]
HPV-45	CA	Gold surface	110 pM	60 s	[47]
HPV-16	EIS	GCE/gold nanosheet	0.15 pM	17 min	[48]
HPV-18	SWV	GCE/carboxyphenyl layer	1.2 × 10^−5^ nM	10 s	[49]
HPV-16	CA	Gold surface	2.39 nM	750 µs	This work

Differential Pulse Voltammetry (DPV); Chronoamperometry (CA); Square Wave Voltammetry (SWV); Cyclic Voltammetry (CV); Electrochemical Impedance Spectroscopy (EIS).
